# Relationship between Helicobacter pylori infection and visceral fat area in diabetic populations: the mediating role of insulin resistance

**DOI:** 10.3389/fcimb.2025.1598223

**Published:** 2025-05-16

**Authors:** Yi Chen, Bingqian Ni, Ningning You, Yahong Chen, Jinshun Zhang

**Affiliations:** ^1^ Departments of Gastroenterology, Taizhou Hospital of Zhejiang Province Affiliated to Wenzhou Medical University, Taizhou, China; ^2^ Departments of Otolaryngology, Taizhou Hospital of Zhejiang Province Affiliated to Wenzhou Medical University, Taizhou, China; ^3^ Home Ward, Taizhou Hospital of Zhejiang Province Affiliated to Wenzhou Medical University, Taizhou, China; ^4^ Health Management Center, Taizhou Hospital of Zhejiang Province Affiliated to Wenzhou Medical University, Taizhou, China; ^5^ Taizhou Hospital of Zhejiang Province, Shaoxing University, Taizhou, China

**Keywords:** Helicobacter pylori, visceral fat area, triglyceride-glucose index, mediation analysis, diabetes

## Abstract

**Background:**

Obesity has become a global health concern, particularly in relation to abdominal fat which is associated with various diseases. The connection between Helicobacter pylori (H. pylori) and obesity is still debated, with limited research on the link between H. pylori and visceral fat. This study aimed to examine the correlation between H. pylori infection and visceral fat area (VFA) among participants.

**Methods:**

A total of 18,076 individuals participated in this study, undergoing assessments of VFA, physical parameter measurements, and serum examinations. Bioelectrical impedance was used to analyze VFA, with the triglyceride-glucose (TyG) index indicating levels of insulin resistance (IR) in the population. Multiple linear regression analysis was utilized to determine the factors influencing VFA, while a generalized additive model was employed to assess potential non-linear associations between the TyG index and VFA.

**Results:**

H. pylori was recognized as a contributing factor to VFA exclusively within diabetic populations. In these populations, a significant nonlinear relationship existed between the TyG index and VFA. Furthermore, mediation analysis indicated that the TyG index acted as a mediator in the relationship between H. pylori and VFA.

**Conclusion:**

H. pylori is closely associated with VFA in individuals with diabetes, with IR serving as a mediating factor in the relationship between H. pylori and VFA.

## Introduction

Obesity is a widespread problem that is on the rise globally, resulting in substantial financial costs (Finkelstein et al., 2009; Afshin et al., 2017). The causes of obesity are multifaceted, encompassing sedentary lifestyle, excessive caloric intake, and additional factors such as psychosocial influences (McAllister et al., 2009). Obesity contributes to higher rates of illness and death due to the link between surplus body weight and elevated chances of developing a range of health issues such as heart diseases, strokes, diabetes, and non-alcoholic fatty liver disease (NAFLD) (Marchesini et al., 2003; Nguyen et al., 2011; Lin and Li, 2021). However, not all excess fat poses the same disease risks (Vilalta et al., 2022). Studies have shown that visceral fat poses a greater danger than subcutaneous fat due to the proteins released by visceral fat cells that can trigger inflammation, atherosclerosis, dyslipidemia, and hypertension (Vilalta et al., 2022; Mao et al., 2024). Moreover, new evidence suggests a connection between visceral obesity and insulin resistance (IR) (Neeland et al., 2019). Despite being the gold standard for assessing IR, the hyperinsulinemic-euglycemic clamp test is not feasible in clinical settings due to practical, ethical, and financial constraints (Du et al., 2014). The triglyceride-glucose (TyG) index, a new clinical surrogate for IR, is gaining research interest for its ease of use, availability, and consistency (Xue et al., 2022; Zhang et al., 2023).

Body Mass Index (BMI), a widely used anthropometric measure, serves as an indicator for fat mass and obesity. (Goossens, 2017). While BMI is valuable for tracking population weight trends and identifying significant potential health risks in individuals with high BMI, it has serious limitations in assessing individual over fatness and fat distribution within the body (Bray, 2023). Bioelectrical Impedance Analysis (BIA) is gaining popularity in large-scale studies for its ease, quickness, and cost-effectiveness as a method for analyzing body composition (Ricciardi and Talbot, 2007; Popiolek-Kalisz and Szczygiel, 2023). BIA offers more precise body composition measurements compared to BMI, establishing it as a more dependable approach for evaluating obesity (Brener et al., 2021).

Recent studies have shown the important impact of gut bacteria on obesity development (Kumari and Kozyrskyj, 2017; Cornejo-Pareja et al., 2019; Scheithauer et al., 2020). Helicobacter pylori (H. pylori) infection is a prevalent gastrointestinal infection that is widely spread worldwide (Zamani et al., 2018). H. pylori strains residing in the gastric mucosa have the capability to secrete vacuolating cytotoxin, urease, and cytotoxins, even when most infected individuals remain asymptomatic (Kusters et al., 2006). Reports suggest that apart from causing gastrointestinal disorders like chronic gastritis, peptic ulcers, and gastric cancer, H. pylori infection can also lead to extra-gastric conditions including metabolic syndrome, cardiovascular diseases, and liver diseases (Kuipers, 1997; Franceschi et al., 2019; Liu et al., 2023).

Diabetes and obesity are both manifestations of metabolic syndrome, with mutual influence, in which IR plays a significant role (Lemieux and Després, 2020). Nevertheless, the connection between H. pylori infection and obesity is still uncertain. Moreover, no research has yet confirmed the correlation between H. pylori infection and visceral fat area (VFA). Hence, this study aims to explore the relationship between H. pylori and VFA in the population, as well as the mediating role of IR.

## Methods

### Research participants

Individuals who received health screenings at Taizhou Hospital from January 2017 to April 2023 were the subjects of this study. To meet the inclusion criteria, participants had to be at least 18 years old, provide full personal details, blood test results, and have completed both a urea enzyme test and bioelectrical impedance analysis. Exclusion criteria included being pregnant, lacking clinical data, having a past of stomach surgery, serious heart conditions (such as individuals with heart pacemakers), and having concurrent cancerous growths. Individuals previously diagnosed with diabetes, who have received antihyperglycemic medication treatment, or whose fasting blood glucose (FBG) levels were ≥7.0 mmol/L or glycated hemoglobin A1c (HbA1c) levels were ≥6.5% at the time of the examination, were classified as the diabetic population. A total of 18,076 individuals participated in this study, including 2,155 diagnosed with diabetes.

### Detection of H. pylori

H. pylori status was assessed through ^13^C or ^14^C urea breath tests during routine health examinations. During the ^13^C breath test, participants rinsed their mouths after fasting, exhaled normally for breath sample collection, swallowed a ^13^C urea capsule, waited for 30 minutes, exhaled for another sample collection, and analyzed both samples using the instrument. In the ^14^C breath test, participants swallowed a ^14^C urea capsule, sat quietly for 15 minutes, exhaled into a gas collection card through a mouthpiece, and underwent testing.

### Detection of visceral fat area

We conducted BIA to measure the participants’ VFA. Participants were required to empty their bladder and bowels in a fasted state before the measurement and remove any metal accessories from their bodies. They stood still for 5 minutes pre-measurement to allow for stable water distribution in the body and adjust to the position for testing. Wearing lightweight clothing, participants stood barefoot on the footpads, grasped the electrodes with both hands aligning their heels with the electrodes at the back of their feet, kept their arms straight without touching the sides of their bodies, and ensured their thighs did not touch.

### Blood biochemical results and parameters

Licensed nurses collected age, gender, medical history, systolic blood pressure (SBP), and diastolic blood pressure (DBP). A fasting blood sample was collected from each participant to measure FBG, HbA1c, total cholesterol (TC), triglycerides (TG), high-density lipoprotein cholesterol (HDL), low-density lipoprotein cholesterol (LDL), total protein (TP), albumin (Alb) levels, and blood creatinine (Cr). The TyG index was calculated using the formula: ln [TG (mg/dL) × FBG (mg/dL)/2] (Guerrero-Romero et al., 2010).

### Statistical analysis

Continuous variables underwent analysis through T-tests and were presented as mean ± standard deviation. Categorical variables were evaluated using chi-square tests and reported as counts and percentages. Multiple linear regression analyses were conducted to further identify factors influencing VFA, with adjustments made for confounding variables on multiple occasions. Generalized additive models (GAM) were applied to investigate any nonlinear associations between the TyG index and VFA. Subsequently, a mediation analysis was conducted to explore the mediating role of the TyG index in the relationship between H. pylori and VFA. The statistical analysis was conducted using R 4.1.3 software. The GAM was implemented through the “mgcv” package, while mediation analysis was executed using the “mediation” package within the R environment.

## Results

### Baseline characteristics

This study involved a total of 18,076 individuals, with participant characteristics detailed in **Table 1**. The H. pylori positive group, as opposed to the H. pylori negative group, exhibited a higher proportion of males, with 67.8% compared to 64.7%. In addition, the H. pylori positive group showed higher levels of TG, Cr, blood pressure, blood glucose, and VFA, while presenting lower levels of TC, HDL, LDL, and Alb.

**Table 1:** Baseline characteristics of all physical examination populations.

Variables	H. pylori-negative (n=12642)	H. pylori-positive (n=5434)	p-value
Gender (n, %)			<0.001
Female	4467 (35.3)	1751 (32.2)	
Male	8175 (64.7)	3683 (67.8)	
Age (year)	46.39 ± 11.15	46.77 ± 10.92	0.033
Triglycerides (mmol/L)	1.88 ± 1.62	1.99 ± 1.86	<0.001
Total cholesterol (mmol/L)	5.23 ± 1.02	5.18 ± 1.02	0.001
High density lipoprotein (mmol/L)	1.40 ± 0.34	1.37 ± 0.32	<0.001
Low density lipoprotein (mmol/L)	2.91 ± 0.76	2.88 ± 0.75	0.004
Total Protein (g/L)	75.16 ± 4.13	74.95 ± 4.08	0.001
Albumin (g/L)	46.41 ± 2.72	46.32 ± 2.71	0.044
Creatinine (μmol/L)	70.73 ± 17.03	71.43 ± 14.90	0.008
Diastolic blood pressure (mmHg)	75.90 ± 11.76	76.84 ± 11.92	<0.001
Systolic blood pressure (mmHg)	125.64 ± 16.70	126.98 ± 17.41	<0.001
Fasting blood glucose (mmol/L)	5.43 ± 1.40	5.52 ± 1.60	0.001
Glycated hemoglobin A1c (%)	5.84 ± 0.89	5.88 ± 0.98	0.029
Visceral fat area (cm^2^)	88.04 ± 32.45	89.19 ± 32.33	0.030

### Differences in VFA in different populations

Given the inconsistent impact of H. pylori infection on diabetic and non-diabetic populations, we conducted a stratified analysis on VFA among these diverse groups. Among individuals without diabetes, the existence of H. pylori infection did not demonstrate a notable variation in VFA levels (**Supplementary Table 1**). Conversely, in the diabetic cohort, individuals in the H. pylori positive group exhibited a notable increase in VFA (P=0.003), as demonstrated in **Table 2**. Similar to the overall population, the H. pylori positive group displayed higher levels of TG, blood pressure, and blood glucose, along with lower HDL levels.

**Table 2:** Baseline characteristics of diabetic populations.

Variables	H. pylori-negative (n=1502)	H. pylori-positive (n=653)	p-value
Gender (n, %)			0.034
Female	374 (24.9)	135 (20.7)	
Male	1128 (75.1)	518 (79.3)	
Age (year)	53.49 ± 9.84	52.22 ± 9.46	0.005
Triglycerides (mmol/L)	2.53 ± 2.34	2.88 ± 2.86	0.005
Total cholesterol (mmol/L)	5.38 ± 1.14	5.39 ± 1.16	0.782
High density lipoprotein (mmol/L)	1.29 ± 0.31	1.25 ± 0.29	0.009
Low density lipoprotein (mmol/L)	3.01 ± 0.81	2.99 ± 0.80	0.702
Total Protein (g/L)	75.24 ± 4.23	75.56 ± 4.28	0.107
Albumin (g/L)	46.08 ± 2.79	46.23 ± 2.85	0.252
Creatinine (μmol/L)	71.66 ± 29.71	70.64 ± 16.97	0.410
Diastolic blood pressure (mmHg)	80.24 ± 11.63	81.87 ± 11.50	0.003
Systolic blood pressure (mmHg)	133.90 ± 17.27	135.54 ± 18.45	0.047
Fasting blood glucose (mmol/L)	7.92 ± 2.70	8.45 ± 3.08	<0.001
Glycated hemoglobin A1c (%)	7.57 ± 1.53	7.79 ± 1.69	0.004
Visceral fat area (cm^2^)	100.89 ± 33.84	106.04 ± 38.24	0.003

### The impact of H. pylori on VFA

**Figure 1:**
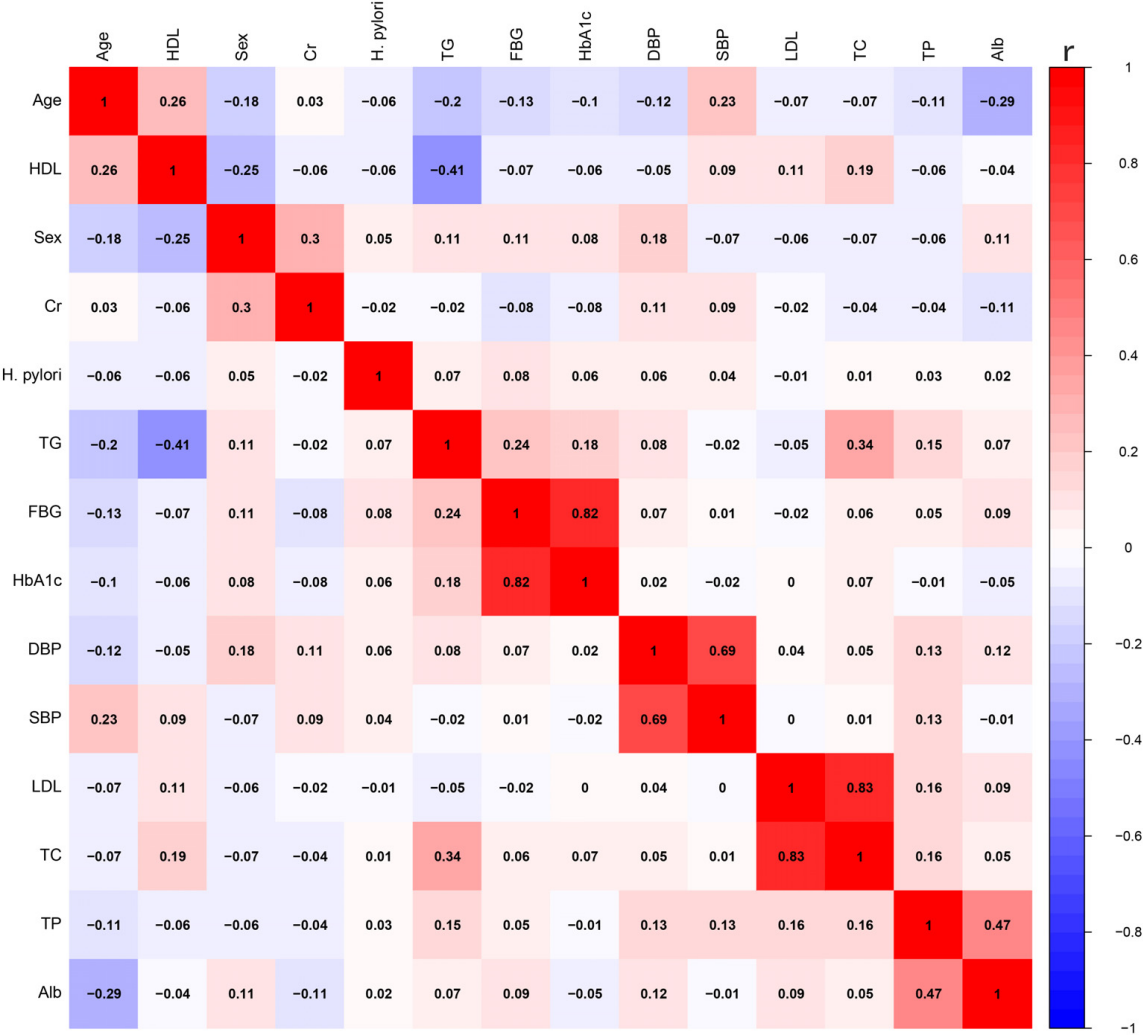
The correlation between various variables. HDL, high-density lipoprotein; Cr, creatinine; TG, triglycerides; FBG, fasting blood glucose; HbA1c, glycated hemoglobin A1c; DBP, diastolic blood pressure; SBP, systolic blood pressure; LDL, low-density lipoprotein; TC, total cholesterol; TP, total protein; Alb, albumin.

To investigate the impact of H. pylori on VFA, we employed multivariable linear regression. After adjusting for lipid profile, blood pressure, blood glucose, Alb, Cr, as well as gender and age, H. pylori infection persisted as a risk factor for VFA in the diabetic population, as shown in **Table 3**. The correlation among variables was illustrated in **Figure 1**.

**Table 3:** Multiple linear regression between H. pylori and VFA.

model	B	SE	β	95% CI	p-value
Model 1	5.539	1.555	0.072	2.489-8.590	<0.001
Model 2	5.149	1.527	0.067	2.154-8.144	0.001
Model 3	4.105	1.482	0.053	1.200-7.011	0.006
Model 4	4.239	1.484	0.055	1.328-7.150	0.004
Model 5	4.132	1.476	0.054	1.238-7.025	0.005

Model 1 is adjusted for age, sex.

Model 2 is adjusted for age, sex, lipids.

Model 3 is adjusted for age, sex, lipids, blood pressure.

Model 4 is adjusted for age, sex, lipids, blood pressure, glucose.

Model 5 is adjusted for age, sex, lipids, blood pressure, glucose, Alb, Cr.

### Relationship between TyG index and VFA

**Figure 2:**
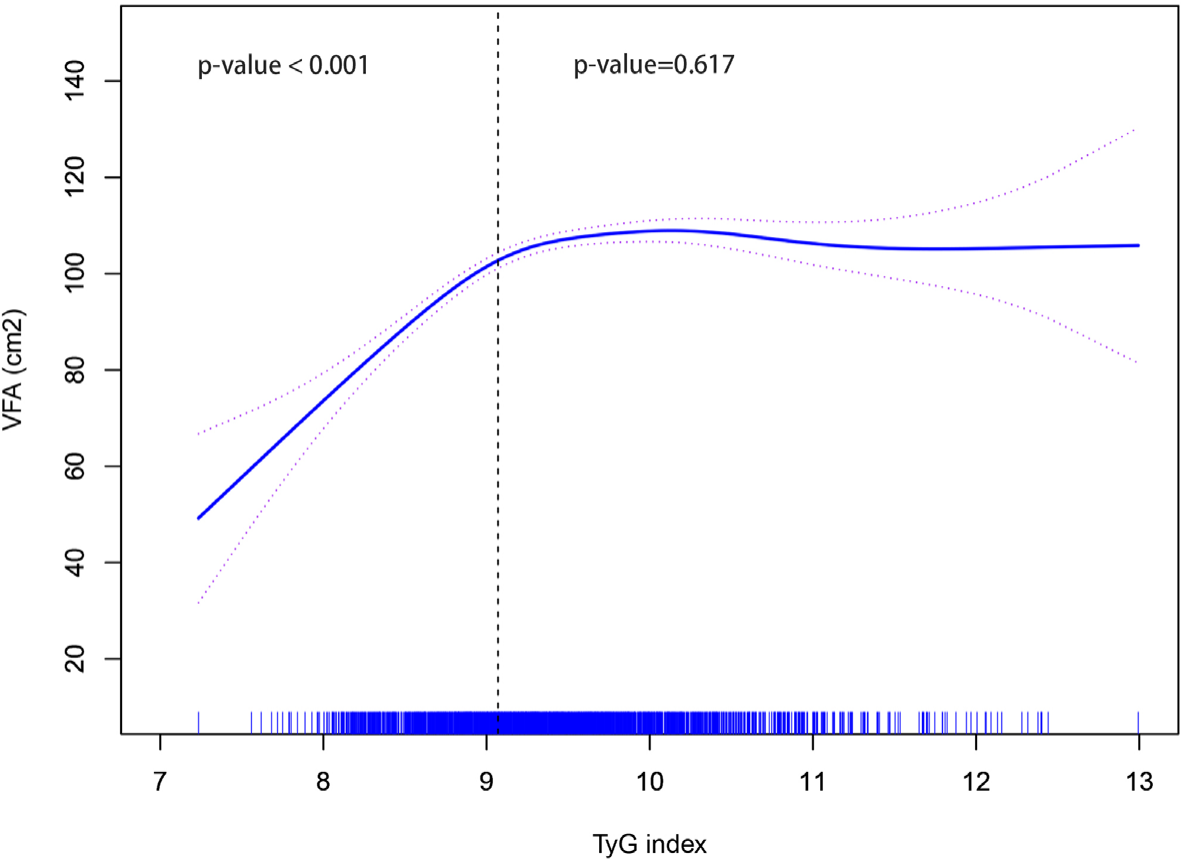
Non-linear relationship between TyG index and VFA. TyG, triglyceride-glucose; VFA, visceral fat area.

Based on the GAM curve, there was a nonlinear relationship between TyG index and VFA. When the TyG index was below 9.07, an increase in the TyG index was linked to an elevation in VFA (P < 0.001). Conversely, if the TyG index exceeded 9.07, no significant correlation existed between the TyG index and VFA (P = 0.617) as illustrated in **Figure 2**.

### Mediation effect analysis

**Figure 3:**
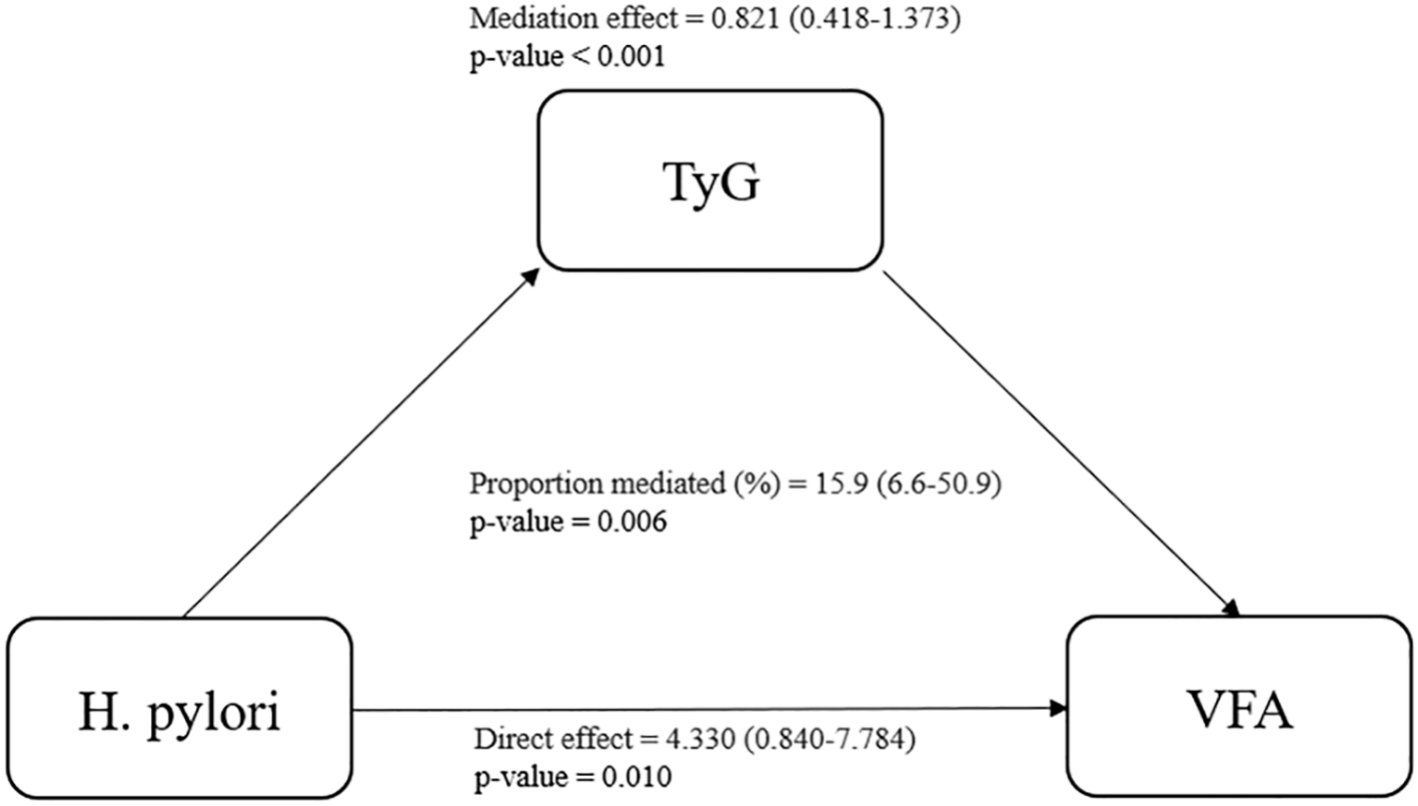
Mediating role of TyG index in the relationship between H. pylori and VFA. TyG, triglyceride-glucose; VFA, visceral fat area. Proportion mediated = mediation effect/(mediation effect + direct effect).

To further investigate the role of the TyG index in the relationship between H. pylori and VFA, we conducted a mediation analysis. The result indicated that the TyG index partially mediated the relationship between H. pylori and VFA, with a mediation effect size of 0.821 (P < 0.001), a direct effect of 4.330 (P = 0.010), and a mediation ratio of 15.9% (P = 0.006), as illustrated in **Figure 3**.

## Discussion

Opinions on the link between H. pylori infection and obesity are divided. In China, a cross-sectional study found a significant correlation between obesity and H. pylori infection (Zhang et al., 2015). Furthermore, H. pylori infection was positively correlated with higher BMI in several studies involving large samples (Suki et al., 2018; Zhang et al., 2020). However, conflicting conclusions have been reported. An analysis of Mendelian randomization found no association between H. pylori infection and obesity (den Hollander et al., 2017). Additionally, in some developed countries, H. pylori infection was negatively correlated with obesity rates (Lender et al., 2014). Nevertheless, there is a lack of research exploring the association between H. pylori and VFA, stratified by diabetes status.

In our research, we discovered that H. pylori infection did not exhibit a significant impact on VFA in the non-diabetic cohort. Yet, individuals with diabetes showed a correlation between H. pylori infection and a rise in VFA. Previous research indicates that when VFA exceeds 100 cm², the risk of obesity-related diseases markedly increases (New criteria for ‘obesity disease’ in Japan, 2002; Kim et al., 2022). Although women typically have less visceral fat than men, they remain at an elevated risk for developing diabetes due to increases in visceral fat (Kim et al., 2022). Our further analysis using multiple linear regression confirmed that H. pylori infection is a significant risk factor for elevated VFA. Infection with H. pylori disturbs the equilibrium of beneficial bacterial species in the stomach lining, causing changes in the human gut microbiome, which is essential for the body’s immune response and metabolic balance (Iino et al., 2018; Lopetuso et al., 2018). In fact, gut microbiota imbalance is closely associated with obesity, diabetes, and metabolic syndrome (Cornejo-Pareja et al., 2019; Dabke et al., 2019; Wu et al., 2023). The progression of diabetes involves chronic low-grade metabolic inflammation, closely intertwined with disturbances in glucose metabolism pathways (Scheithauer et al., 2020). Adipose tissue not only serves as an important energy storage organ but also is significantly associated with inflammation and IR (Gregor and Hotamisligil, 2011; Wensveen et al., 2015; Lu et al., 2019). Previous studies have demonstrated that IR can promote the accumulation of visceral fat and exacerbate the progression of diabetes through various inflammatory factors and metabolic pathways (de Mutsert et al., 2018; Yaribeygi et al., 2019; Lee et al., 2022). This study established a significant correlation between IR and VFA in diabetic populations. The relationship between H. pylori and IR has been reported in earlier studies, with some suggesting that H. pylori infection may contribute to the development of IR by activating chronic inflammatory responses, disrupting insulin signaling pathways, increasing oxidative stress levels, and altering gastrointestinal hormone secretion (Aslan et al., 2006; Azami et al., 2021). Our results confirmed that IR plays a significant mediating role in the relationship between H. pylori and VFA, indicating that IR may be one mechanism by which H. pylori affects visceral fat deposition. Notably, diabetic populations typically exhibit higher levels of IR, and H. pylori may exert its effects on visceral obesity specifically under this pathological state. Consequently, early intervention targeting H. pylori infection, particularly eradication of H. pylori, may hold significant clinical implications for reducing visceral fat accumulation in diabetic populations. Although H. pylori secretes various virulence factors, the specific factors affecting VFA remain unidentified. VFA is closely linked to a range of chronic diseases, and if not addressed, its continued accumulation may have serious health consequences. Therefore, future large-scale longitudinal studies and investigations into the biological mechanisms are needed to further elucidate the precise pathways through which H. pylori infection contributes to metabolic regulation in diabetic individuals and to explore the potential benefits of H. pylori eradication therapy in diabetic patients with visceral obesity.

Our study validated the influence of H. pylori infection on VFA in people with diabetes. However, this study still has shortcomings. Firstly, being single-centered, whether the research findings can be generalized to other regions necessitates further validation through multicenter studies. Besides, the study lacks examination of factors such as lifestyle, psychosocial influences, and socioeconomic status, all of which are crucial influences on visceral obesity. Moreover, the specific mechanism by which H. pylori infection solely affects the VFA in individuals with diabetes remains unclear, demanding prospective studies and additional biological experiments for verification.

## Conclusion

In diabetic populations, there is a significant association between H. pylori infection and VFA, with IR serving as a mediating factor in the association between H. pylori and VFA.

## Data Availability

The raw data supporting the conclusions of this article will be made available by the authors, without undue reservation.
